# The Relationship Between Benign Paroxysmal Positional Vertigo and Vitamin D

**DOI:** 10.7759/cureus.26068

**Published:** 2022-06-18

**Authors:** Ali Seyed Resuli, Ahmet Bedir, Abdülkadir Özgür

**Affiliations:** 1 ENT, İstanbul Yeni Yüzyıl University, Faculty of Medicine, Gaziosmanpaşa Hastanesi, İstanbul, TUR

**Keywords:** canalithiasis, cupulolithiasis, otoconia, vitamin d, vertigo

## Abstract

Introduction

Benign paroxysmal positional vertigo (BPPV) is a type of vertigo and its signs are short-time, severe attacks that occur in certain head and body positions. Recent studies have revealed that vitamin D deficiency correlates with BPPV and this is explained by cupulolithiasis and canalithiasis theories.

Method

In the present study, levels of serum vitamin D in the patients who were diagnosed as BPPV and those in the control group consisting of healthy individuals were investigated. In addition, it was examined whether vitamin D is influential on the rates of BPPV types. In our study, 258 patients who were diagnosed with BPPV after detailed ear-nose-throat and neurology examinations were examined. We compared the control group according to their ages, genders, and levels of vitamin D. In addition, we divided the BPPV group into two sub-groups according to their vitamin D levels (20-30 ng/ml and 20 g/ml lower), and each was compared by calculating vertigo types and ratios.

Results

The BPPV group included 187 females and 71 males, and their mean age was 43.70 ± 15.44. The control group consisted of 65 females and 35 males, and the mean age of this group was 44.63 ± 15.42. The mean vitamin D levels of the females and males were 18.42 ± 5.07 and 19.82 ± 5.11, respectively, in this study. On the other hand, the mean vitamin D levels of healthy females and males were found to be 30.88 ± 10.74.

Conclusion

Our study found that the vitamin D levels of the individuals in the BPPV group were statistically significantly lower than those of the individuals who were in the control group. However, it was observed that vitamin D did not affect the rate of vertigo subtypes.

## Introduction

Benign paroxysmal positional vertigo (BPPV) is considered one of the primary causes of peripheral vertigo [1,2]. BPPV is a disease in which changes in body position result in clinical symptoms like vertigo, vomiting, and dizziness. The disease is diagnosed through horizontal nystagmus and clinical symptoms [3-5].

BPPV occurs when otoconia are dislodged from the macula of the utricle or saccule and enter the semicircular canal (SSC) or attach to the cupula [6,7]. Otoconia consist of CaCO_3_ and glycoprotein crystals, which are its main elements and are linked to hair cells through protein fibres. Active calcium metabolism processes in the vestibular organ form the otoconia crystals [8]. Otoconia crystals include a central nucleus whose main components are organic glycoproteins with low calcium (Ca) levels. The crystals are surrounded by inorganic peripheral areas with minerals that consist mainly of CaCo_3_ with high Ca levels [9].

It is known that Ca channel proteins linked to vitamin D in the epithelium are present in the Ca metabolism of the vestibular organ. Furthermore, studies have examined the relationship between Ca-related diseases and BPPV [9]. In addition to studies indicating that BPPV patients have lower levels of vitamin D than controls, and some case studies have shown severe vitamin D deficiency in patients who chronically suffer from BPPV recurrence [10-12].

Vitamin D is synthesized in the skin and the organ in which it is transformed into 25-OH vitamin D in the liver. Compared with other metabolites, serum concentrations of 25-OH vitamin D are the highest in humans [9]. In this study, we examined the impact of serum 25-OH vitamin D concentrations on the recurrence of BPPV and its types. In healthy Turkish people, the serum level of vitamin D is 30-50 ng/ml [13,14]. A vitamin D level between 20 and 30 ng/ml is considered low, and below 20 ng/ml is considered very low [15].

## Materials and methods

This study included 258 patients who presented to our study centers between 2010 and 2018. They complained of dizziness and vomiting, and they were diagnosed with recurrent BPPV (a minimum of five episodes of vertigo per week). The control group comprised healthy individuals whose demographic characteristics were comparable to those of the patients in the BPPV group.

**Figure 1:**
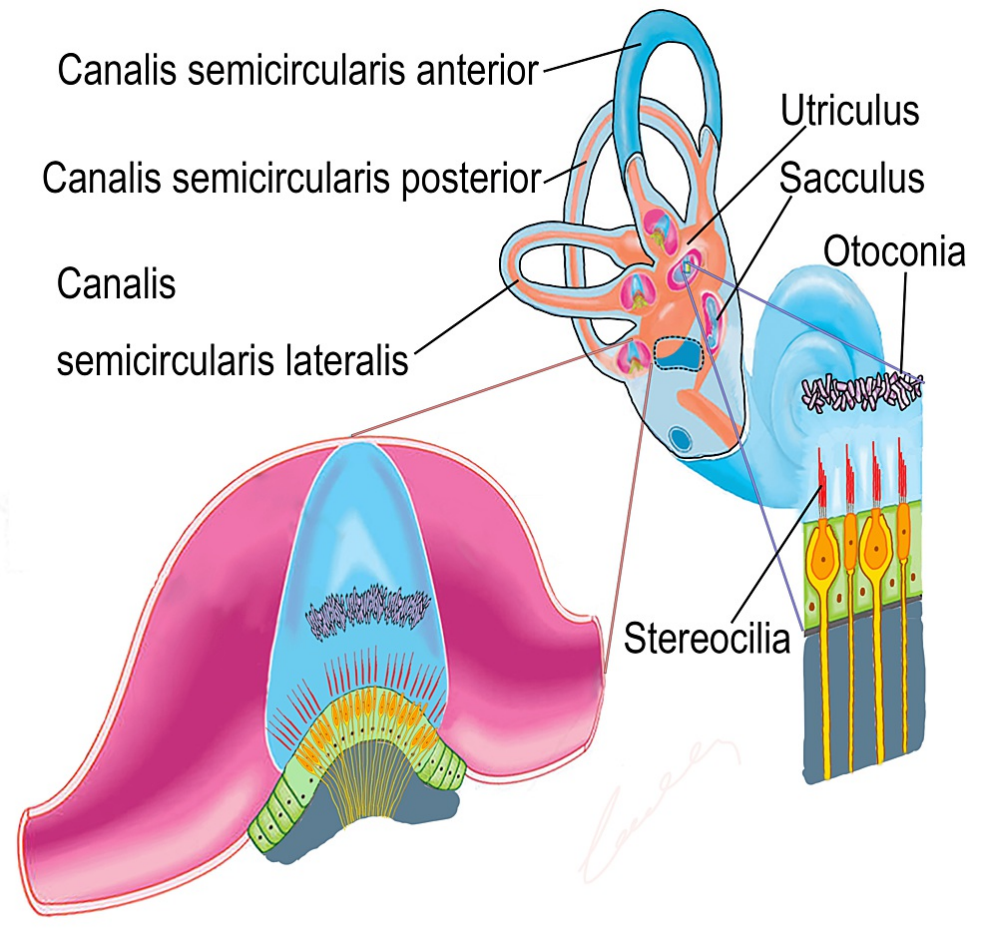
Vestibular system.

BPPV was diagnosed based on the diagnostic criteria of the American Society of Otolaryngology and Head and Neck Surgery [3]. Patients in both groups were neurologically and ontologically evaluated and cerebral-cerebellar tests were conducted. The Dix-Hallpike (for posterior SSC) [16], supine roll (for lateral SSC) [17], and cephalic hyperextension (for anterior SSC) [18] tests were performed on all patients to investigate the horizontal nystagmus and the type of BPPV (Figure 1). The patients were treated using the Epley repositioning manoeuvre for a posterior semicircular canalolith [19], the rotation manoeuvre for a horizontal semicircular canalolith [20], and the Yacovino manoeuvre for an anterior semicircular canalolith [21]. The manoeuvres were performed by the patients two to three times each day. The patients returned for follow-up three days after the repositioning manoeuvres and were evaluated by an otolaryngologist physician who was experienced in the field. The disappearance of signs and nystagmus were the criteria for a full recovery.

All patients’ serum 25-OH vitamin D levels were measured using a chemiluminescence immunoassay (CIA) (Beckman Coulter Access 2). We classified the patients into two groups: (i) BPPV (patients who had the disease) and (ii) non-vertigo (patients who were healthy). We then examined whether serum vitamin D levels influenced the frequency of different BPPV types. Patients' age, gender, and serum vitamin D concentration were recorded, and these values were then analysed according to the groups.

This study was approved by the ethics committee of the İstanbul Yeni Yüzyıl University. The ethics committee file number is 2018/8.

Data were analysed using SPSS version 21.0 (IBM Corp., Armonk, NY), and descriptive statistics were presented as frequency, arithmetic mean, standard deviation, percentage, or minimum and maximum values. The data did not show a normal distribution; therefore, nonparametric tests were utilised in the analysis. The mean values of the two independent groups were compared using the Mann-Whitney U test, and two independent groups were compared using the Chi-square test. In the relationship analysis of BPPV, the binary logistic regression coefficient was calculated. A significance level of p=0.05 was applied in the study. The BPPV group was further divided into two subgroups based on their vitamin D levels: (i) patients whose vitamin D levels were below 20 ng/ml and (ii) patients whose vitamin D levels were between 20 and 30 ng/ml. In addition, whether there was a significant difference between these two subgroups in terms of vertigo subtype was examined.

## Results

The BPPV group included 187 females and 71 males, and their mean age was 43.70 ± 15.44 years (range: 17-87 years). The control group consisted of 65 females and 35 males, and the mean age of this group was 44.63 ± 15.42 years (min-max) (Table 1).

The mean vitamin D levels of the females and males were 18.42 ± 5.07 ng/ml and 19.82 ± 5.11 ng/ml, respectively. In contrast, the mean vitamin D levels of healthy females and males were 30.88 ± 10.74, respectively.

**Table 1:** Descriptive characteristics of patients with BPPV and healthy patients. BPPV: benign paroxysmal positional vertigo.

Variables	BPPV (n=258)	Control (n=100)
Age	43.70 ± 15.44	44.63 ± 15.42
Female	187	65
Male	71	35
Vitamin D	18.80 ± 6.10	30.74 ± 8.53

No difference between the BPPV and control groups regarding age according to the Mann-Whitney U test results (p=0.50). However, it was found that vitamin D levels were significantly lower in the BPPV group than in the healthy control group. Moreover, the differences between the groups were statistically significant (p=0.00) (Table 2).

**Table 2:** The relationship between the groups based on the Mann-Whitney U test. BPPV: benign paroxysmal positional vertigo. *Statistically significant.

	Mean rank	Mean rank		
Variables	BPPV (N=258)	Control (n=100)	Mann-Whitney U test	P
Age	177.24 ± 15.44	185.34 ± 15.42	12316.00	0.502
Vitamin D	140.44 ± 6.10	280.27 ± 8.53	2823.500	0.000*

According to the results of the Chi-square analysis (p>0.05), no significant differences were observed between the groups regarding gender (Table 3).

**Table 3:** The relationship between the groups in terms of gender. BPPV: benign paroxysmal positional vertigo.

Gender	BPPV	Control	Test	P
Female	187 (72.5%)	65 (65%)	1.93	0.164
Male	71 (27.5%)	35 (35%)		

Bilateral logistic regression analyses were performed to confirm that the factors affecting BPPV included all the variables in Table 1. It can be argued that vitamin D and age contributed to the emergence of BPPV (p<0.05); however, no significant association was observed with vertigo types (p>0.05). In addition, the contribution of vitamin D to the development of BPPV was noted to be 1.2 times (p<0.05, Exp (B) 1.25) (Table 4).

**Table 4:** Binary logistic regression’s analysis of the BPPV group. SE: standard error and BPPV: benign paroxysmal positional vertigo. *Statistically significant.

Variables	B	SE	Sig	Exp (B)
Ages	−0.011	0.012	0.358	0.989
Gender	−0.758	0.358	0.032*	0.469
Vitamin D	0 .225	0.041	0.000*	1.252
Posterior canal			1.000	
Lateral canal	−240.688	40,619.405	0.995	0.000
Anterior canal	−347.854	53,216.166	0.996	0.000
Multiple canal	−235.437	45,102.772	0.996	0.000

Table 5 shows the descriptive statistics of vertigo types and vitamin D levels, which were D<20 and D 20-30 in the BPPV group. Here, the mean scores of the former and latter groups were 14.47 ± 3.32 and 24.40 ± 3.49, respectively. According to the findings, the posterior canal was the most common and the anterior canal was the least common type of vertigo (Table 5).

**Table 5:** Descriptive statistics of BPPV group (n=258). BPPV: benign paroxysmal positional vertigo.

	Vitamin D<20	Vitamin D 20-30	Test	P
Age	43.65 ± 16.09	43.63 ± 14.67	7860.50	0.06
Female	115	72	3.31	0.06
Male	33	38		
Mean of vitamin D	14.47 ± 3.32	24.40 ± 3.49	0.00	0.00
Posterior canal	130 (87.2%)	92 (84.4%)	0.79	0.85
Lateral canal	13 (8.7%)	13 (11.9%)		
Anterior canal	4 (1.5%)	3 (2.8%)		
Multiple canal	2 (5%)	1 (0.9%)		

No significant relationship was found between vertigo type and vitamin D level (Table 6).

**Table 6:** The relationship between vitamin D3 and vertigo in the BPPV group. BPPV: benign paroxysmal positional vertigo.

Variable			Mann-Whitney U	P
	D<20 (N=148)	D 20-30 (n=110)		
Vertigo type	126.81	128.49.34	7719.00	0.761

## Discussion

In this study, we examined the effects of serum vitamin D levels in patients who were diagnosed with recurrent BPPV. First, we investigated the pathophysiology of the disease and vitamin D. There are three semi-circular canals (anterior, posterior, and horizontal) and two otoliths (utricle and saccule) in the vestibular part of the membranous labyrinth. The source of the calcium carbonate crystals (otoconia) that are responsible for BPPV is the macula of the saccule [22]. Canalithiasis refers to the displacement of otoconia located within the gelatinous membrane in the macula into the semicircular canals. Cupulolithiasis refers to the adherence of these particles to the cupula of the semi-circular canals. BPPV pathophysiology is thought to be caused by these two conditions [23].

Karataş et al. found that osteoporosis and vitamin D deficiency were highly prevalent in the overall population, and the situations where BPPV coexisted with osteoporosis with vitamin D deficiency were incidental [24]. In another study, Kahraman et al. determined that vitamin D deficiency and low ionised Ca levels could result in BPPV not only in patients diagnosed with osteoporosis but in all patients [25].

Büki et al. showed that serum levels of vitamin D in patients with BPPV were similar to those of the rest of the population and that these levels were much lower in patients who experienced recurrent BPPV than in those who experienced their first BPPV attack [11].

Talaat et al. argued that low levels of vitamin D were only related to BPPV development; however, lower levels of vitamin D played a role in the recurrence of the disease [26]. In addition, another study conducted by Talaat et al. established that treatment of severe vitamin D deficiency may influence the increase in BPPV recurrence [27].

Posterior channel BPPV is regarded as the most frequently encountered type of BPPV; it is responsible for 90% of all BPPVs [28]. According to the American Society of Otolaryngology and Head and Neck Surgery, the diagnostic criteria of posterior channel BPPV are a history of recurrent vertigo attacks following head movements, nystagmus, and vertigo triggered by the Dix-Hallpike manoeuvre.

Lateral (horizontal) channel BPPV is another type of BPPV that accounts for nearly 10% of all BPPVs [29]. In many cases, the history of lateral channel BPPV is similar to that of posterior channel BPPV. However, in patients with a negative Dix-Hallpike test, lateral channel BPPV should be considered, and a supine roll test should be performed [30,31].

Anterior channel BPPV is very rare; it accounts for 1-2% of BPPVs. It typically appears with downbeat nystagmus. However, such nystagmus can also be seen in cerebellar and brainstem lesions. Therefore, the diagnosis of anterior channel BPPV is important. The cephalic hyperextension test is used for the diagnosis of anterior channel BPPV [18].

In our study, the BPPV and control groups were similar regarding age, gender, seasonal factors, skin colour, habit of dressing, and diet. In our case, we found that vitamin D levels were significantly lower in the BPPV group than in the control group. Women in the BPPV and control groups had lower vitamin D levels than men, and the levels of sunlight that women were exposed to were lower than men due to their traditional style of dressing. In addition, we divided the BPPV group into two subgroups based on their levels of vitamin D: (i) those with low (20-30 ng/ml) and (ii) those with very low (below 20 ng/ml) serum vitamin D. We evaluated the frequency of each vertigo type among these subgroups; no significant association was found between vertigo type and vitamin D deficiency. The limitations of our study are not checking serum Ca levels in the study group and also not investigating the osteoporosis levels of the patients in this group. In further studies, the relationship between serum Ca, osteoporosis and BPPV should be investigated in high patient groups.

## Conclusions

The present study found a significant difference in the serum vitamin D levels of individuals in the BPPV and control groups. However, no significant association was observed between vitamin D levels and vertigo type.
